# Evolution of the dense packings of spherotetrahedral particles: from ideal tetrahedra to spheres

**DOI:** 10.1038/srep15640

**Published:** 2015-10-22

**Authors:** Weiwei Jin, Peng Lu, Shuixiang Li

**Affiliations:** 1 Department of Mechanics and Engineering Science, College of Engineering, Peking University, Beijing 100871, China

## Abstract

Particle shape plays a crucial role in determining packing characteristics. Real particles in nature usually have rounded corners. In this work, we systematically investigate the rounded corner effect on the dense packings of spherotetrahedral particles. The evolution of dense packing structure as the particle shape continuously deforms from a regular tetrahedron to a sphere is investigated, starting both from the regular tetrahedron and the sphere packings. The dimer crystal and the quasicrystal approximant are used as initial configurations, as well as the two densest sphere packing structures. We characterize the evolution of spherotetrahedron packings from the ideal tetrahedron (*s* = 0) to the sphere (*s* = 1) via a single roundness parameter *s*. The evolution can be partitioned into seven regions according to the shape variation of the packing unit cell. Interestingly, a peak of the packing density Φ is first observed at *s* ≈ 0.16 in the Φ-*s* curves where the tetrahedra have small rounded corners. The maximum density of the deformed quasicrystal approximant family (Φ ≈ 0.8763) is slightly larger than that of the deformed dimer crystal family (Φ ≈ 0.8704), and both of them exceed the densest known packing of ideal tetrahedra (Φ ≈ 0.8563).

Particle packing problems have a long history which can be traced back to the dawn of civilization. Dense particle packings have received much attention as models for a variety of condensed matter systems, including glasses, crystals, heterogeneous materials, and granular media^1^,^2^,^3^,^4^. The question that “how one can arrange most densely in space an infinite number of equal solids of given form, e.g., spheres with given radii or regular tetrahedra with given edges” was presented in the Hilbert’s 18^th^ problem^5^. Sphere and tetrahedron are two special and interesting cases of this significant problem. The densest packing of congruent spheres, known as the Kepler conjecture, is the face-centered cubic (FCC) packing or the hexagonal close packing (HCP) arrangement^5^ with the same packing density of about 0.7405, whereas the densest packing of tetrahedra is still sealed. The densest known packing of tetrahedra is the dimer crystal^6^ (DC) configuration, which is composed of four tetrahedra forming two dimers per unit cell, with a density of about 0.8563. A quasicrystal approximant^7^ (QA), whose density is only slightly less than that of the densest dimer crystal, is composed of 82 tetrahedra per unit. The two configurations may be the two possible candidates for the densest packing of tetrahedra, i.e., the solutions for the tetrahedron case of the Hilbert’s 18^th^ problem.

The packing of spheres has been extensively studied for its simplicity, while investigations of non-spherical particles are much less^8^. Packing behavior is close related to the shapes of the particles, which are often non-spherical in nature. Recently, predictive frameworks of non-spherical particles have been made in the geometrical model of the mean field theory by Baule *et al.*^8^,^9^, which showed a good agreement with the random close packing densities of axisymmetric particles in simulations, and of directional entropic forces by van Anders *et al.*^10^ through local dense packing. As a typical type of non-spherical particles, tetrahedra have attracted particular attention for their simplicity and the lack of inversion symmetry^11^. Many researchers have studied the ordered, disordered and the special maximally random jammed state of tetrahedra^6^,^7^,^8^,^9^,^10^,^11^,^12^,^13^,^14^,^15^,^16^,^17^,^18^,^19^,^20^,^21^,^22^,^23^,^24^,^25^,^26^,^27^. Furthermore, deformations of tetrahedra have been investigated to see the shape effect on packing structures and properties. Jiao *et al.*^28^ analytically constructed the densest known packing of Archimedean truncated tetrahedra with a packing density of 0.9952. Chen *et al.*^29^ further explored the phase behavior of systems of truncated tetrahedra, and obtained the freezing- and melting-point packing densities for the liquid-solid transition, and achieved the range of coexistence densities for the solid-solid transition. The thermodynamic properties of a truncated tetrahedron family varying from tetrahedra to octahedra were studied by Damasceno *et al.*^30^, and they observed several atomic crystal isostructures and found a new space-filling polyhedron. Recently, tetrahedra continuously deformed via vertex and/or edge truncations were explored by Chen *et al.*^31^, which showed that small imperfections in the particle shape may lead to completely different packing structures. All these aforementioned deformations of tetrahedra were obtained by truncating particles with planes. They are convex shape families transforming among tetrahedron, octahedron and cube. Kallus and Elser^32^ studied the dense crystal structures of physical tetrahedra and found four structures as candidates for the optimal packing at different asphericities. Zhao *et al.*^25^ investigated the rounded corner effect on the random packing of spherotetrahedra, and the random packing density showed a continuous decrease trend with the increase of roundness. Although the physical tetrahedra and spherotetrahedra are two different deforming patterns of tetrahedra, they both reveal the continuous deformation from sphere to ideal tetrahedron, which is important for studying the evolution of packing properties from sphere to non-spherical particles.

Notably, real tetrahedral particles from the nanoparticles^33^,^34^,^35^ synthesized at micro-scale to the macroparticles (dice) used in experiments^18^,^19^,^20^,^25^ all have rounded edges and vertices. Yet, the effect of rounded corners on the packing structure and on the maximum packing density of particle systems is still vague. Hence, we investigate the rounded corner effect on the dense packings of spherotetrahedral particles in this work. The dense packing of spherotetrahedral family is also helpful for the detection of the densest packing of ideal tetrahedra. Two dense tetrahedron packings, i.e., the dimer crystal and the quasicrystal approximant, as well as the two dense sphere packings, i.e., the face-centered cubic and the hexagonal close packings, are used as the original structures for evolving from the ideal tetrahedron to the sphere. A single roundness parameter *s* is then introduced to measure the roundness degree of a particle. We analyse the shape variation of the packing unit cell, which partitions the packing evolution into seven regions, by calculating the lengths of the three lattice vectors of the unit packing cell and the angles between them. The existence of rounded corners causes rearrangements of particles, which leads to the variety of packing densities. However, packing density is a macroscopic factor, while local particle arrangement is a microscopic property. Hence, we introduce a new local parameter δ_c_ to characterize approximately the effects of local particle structures and rounded corners. The parameter weights the center separating distance among particles for removing overlaps caused by virtual regrown sharp corners. It shows good linear correlations with the number density in all the seven partitioned regions. Furthermore, an increase of the packing density is observed in the Φ-*s* curves when the corners of tetrahedra begin to round. The maximum density of the deformed quasicrystal approximant family (Φ ≈ 0.8763) is slightly larger than that of the deformed dimer crystal family (Φ ≈ 0.8704), and both of them exceed the densest known packing of ideal tetrahedra (Φ ≈ 0.8563).

## Methods

### Model and algorithms

**Figure 1:**
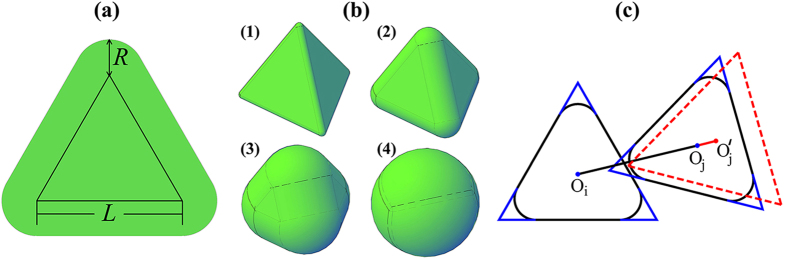
(**a**) The definition of a spherotetrahedron in two-dimensional schematic diagram. (**b**) Numerical models of four spherotetrahedra with the roundness ratio *s* of (1) 0.16, (2) 0.5, (3) 0.84 and (4) 0.98. (**c**) The definition of the local parameter δ_c_ in two-dimensional schematic diagram. If two particles *i* and *j* have overlaps when they regrow shape corners, particle *j* is translated along ***O***_***i***_***O***_***j***_ until no overlap exists between them regardless of other overlapping pairs of particles. The translation vector is ***O***_***j***_***O***_***j***_′.

Considering the existence of rounded corners of real tetrahedral particles, we use the spherotetrahedron model^25^ in this work to imitate the rounded corner of real particles. A spherotetrahedron can be mathematically treated as a set of points with the distance to an inner tetrahedron smaller than a given value *R*, as illustrated in Fig. 1(a). We define the roundness ratio of a spherotetrahedron as


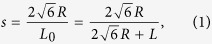


where *L* is the edge length of the inner tetrahedron, and *L*_*0*_ is the edge length of a tetrahedron having the same sized inscribed sphere with the spherotetrahedron. The family of spherotetrahedra is then parametrized by the ratio *s* and ranges from the ideal tetrahedron (*s* = 0) to the sphere (*s* = 1). Hence, the spherotetrahedron model can be applied to study the rounded corner effect and explore the evolution from the tetrahedron packing to the sphere packing. Figure 1(b) shows four samples of spherotetrahedra with different *s* values.

In this work, the adaptive shrinking cell (ASC) algorithm^14^,^15^,^24^,^29^ and the relaxation algorithm^25^,^26^,^27^ are used to generate the packings of spherotetrahedra with different roundness ratios. The dimer crystal (DC) and the quasicrystal approximant (QA) are applied as the initial configurations to evolve from the ideal tetrahedron packing to the sphere packing by increasing the roundness ratio *s* gradually, while the FCC and the HCP structures are used as the initial structures for the inverse evolution from the sphere packing to the ideal tetrahedron packing.

### Local parameter δ_c_

In this work, we attempt to analyse the packing property from a local perspective to see the connection between the local structure of a particle system and the packing boundary of a unit cell. We define a parameter δ_c_ to weight the local particle center separating distance caused by virtual regrown sharp corners, as demonstrated in Fig. 1(c). This local parameter δ_c_ is given by


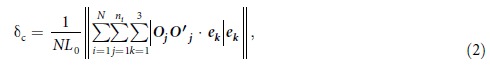


where ***e***_***k***_ (*k* = 1, 2, 3) is the base vector of the Eulerian coordinates and *n*_*i*_ is the number of overlapped particles with particle *i* when all particles regrow shape corners. The vector ***O***_***j***_***O***_***j***_′ is the separating vector obtained by translating particle *j* along the direction ***O***_***i***_***O***_***j***_ until no overlap exists, where *O*_*i*_ and *O*_*j*_ are the centers of the two overlapped particles *i* and *j*, respectively. The local parameter δ_c_ is determined by the local topological structures (contact types) of particles and the roundness ratio *s*. Consequently, we use the parameter δ_c_ to characterize the effects of local particle arrangements and rounded corners.

## Results

### Packing density Φ VS roundness ratio *s*

**Figure 2:**
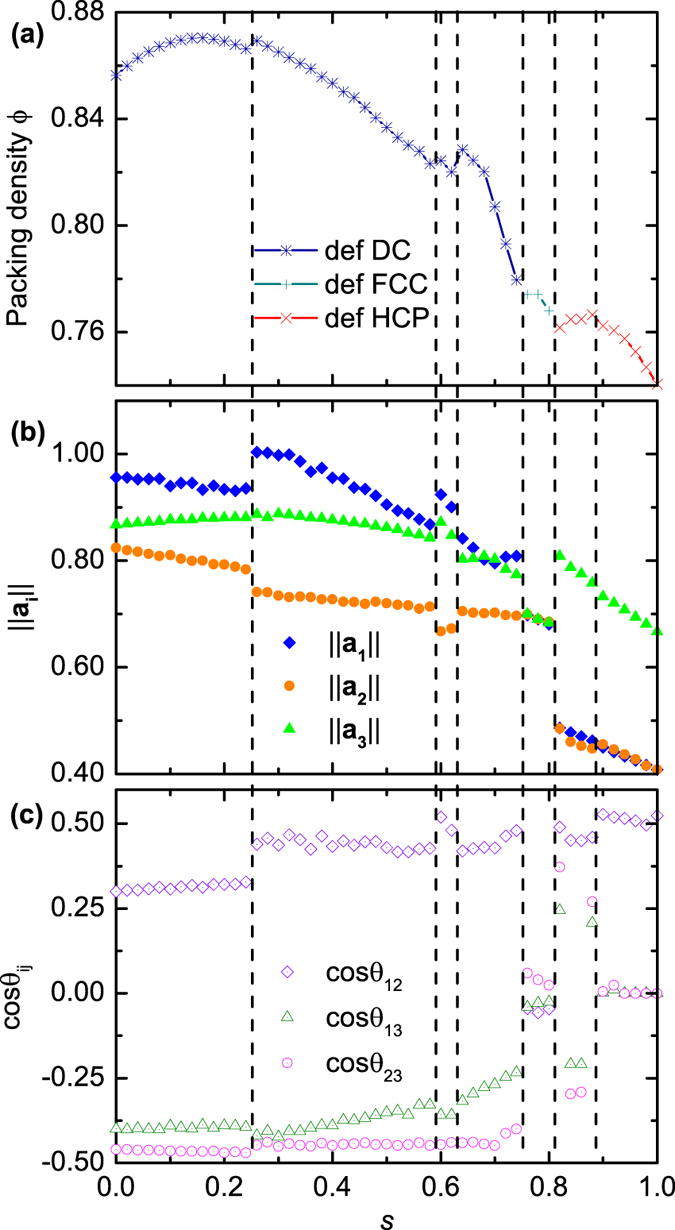
(**a**) The packing density Φ for the dense packing of spherotetrahedra as a function of *s*. (**b**) The length ||**a**_**i**_|| (*i* = 1, 2, 3) of the three lattice vectors **a**_**i**_. (**c**) The cosine of the angles θ_ij_ (*i* < *j* = 1, 2, 3) between the three **a**_**i**_. Grey vertical dash lines partition the *s* domain into seven regions.

**Figure 3:**
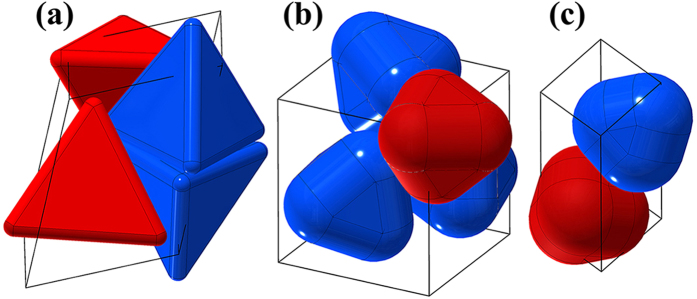
Illustration of three dense evolution families with periodic boundaries. (**a**) Deformed DC at *s* = 0.26 with four particles participating in two inverted pairs per unit cell. (**b**) Deformed FCC at *s* = 0.78 with one particle located on a vertex of the unit cell and the other three sited on the centers of three cell surfaces approximately. (**c**) Deformed HCP at *s* = 0.88 with two particles per unit cell forming analogue of hexagonal system.

Figure 2(a) gives the relationship between the density Φ of the dense spherotetrahedron packings, which are evolved from the initial DC, FCC and HCP structures, and the roundness ratio *s*. The results are the densest structures obtained from our simulations at each roundness ratio and may not be the theoretical upper bounds. Optimizing these structures might lead to denser packings. The packing evolution can be then mainly classified into three families according to the topological structure of particles, i.e., the deformed dimer crystal (def DC), deformed FCC (def FCC), and deformed HCP (def HCP) families, as illustrated in Fig. 3. The evolution from the QA structure is discussed afterwards considering its great difference in particle amount and boundary shape with the other three structures. It can be seen that the packing density curve is discontinuous with several breaks. Following Ref. 36we use the lengths ||**a**_**i**_|| of and the angles θ_ij_ (*i* < *j* = 1, 2, 3) between the three lattice vectors **a**_**i**_ of the unit packing cell to analyse the results, as shown in Fig. 2(b,c). The curve of the packing evolution can be subdivided into seven regions based on the discontinuities of ||**a**_**i**_|| and cosθ_ij_. It indicates that the breaks of the cell shape variation lead to the discontinuities of the packing density curve.

Figure 3 shows three typical families during the increase of the roundness ratio. When the roundness ratio is small, as shown in Fig. 3(a), particles in the def DC family are composed of two inverted pairs. The two particles in each pair are face to face contacted with a twist angle. This angle increases when the roundness ratio becomes larger. The structure jumps from the def DC to the def FCC structure at *s* = 0.75 and finally to the def HCP at *s* = 0.81. Particles in the def FCC family pack with one located on a vertex of the unit cell and the other three sited around the centers of three cell surfaces, respectively, as shown in Fig. 3(b). The cell boundary of the def FCC family is slightly sheared compared to that of the FCC lattice. Figure 3(c) shows a def HCP example, which contains two particles per unit cell forming analogue of hexagonal system. The entire evolution reveals the diversity of packing behaviors from the tetrahedron packing to the sphere packing.

### Local analysis

The packing density is a macroscopic and primary property of a packing structure. It is the most easily measured value of a packing system. The packing density Φ of a hard particle system can be formulized as


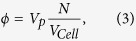


where *N* is the particle amount, *V*_*p*_ denotes the particle volume, which can be written as a function of *L* and *R* analytically, and *V*_*Cell*_ is the packing cell volume. The packing density is then separated into two factors, the particle volume *V*_*p*_ and the number density *N/V*_*Cell*_.

**Figure 4:**
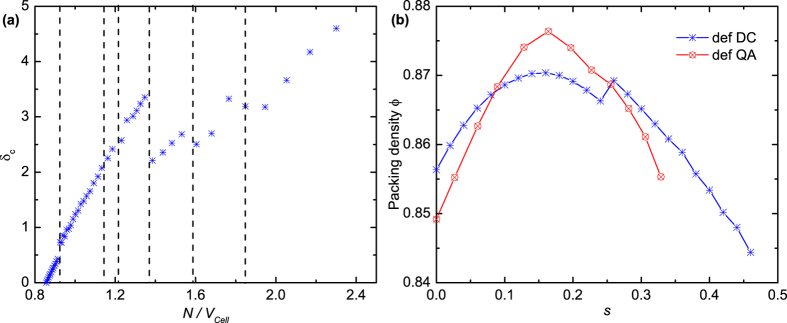
(**a**) The relationship between the local parameter δ_c_ and *N/V*_*Cell*_ for the def DC, def FCC, and def HCP families. The seven regions partitioned by the grey vertical dash lines are consistent with that in Fig. 2. (**b**) The packing density Φ for the dense packing of spherotetrahedra of the def DC and the def QA families as a function of *s*.

The relationship between the local parameter δ_c_, which is defined in the Methods section, and the global parameter *N/V*_*Cell*_ is shown in Fig. 4(a). Note that a linear correlation between the two parameters is observed in each of the seven subdivided regions. The discontinuity of the δ_c_ curve is consistent with that of the ||**a**_**i**_|| and cosθ_ij_ of the packing cell. It demonstrates that the macroscopic packing cell and the microscopic packing structures are close connected with a linear relationship. Nevertheless, it is inappropriate to say that the local packing topologies determine the global packing cell or the global boundary condition governs the local structures. The effect between the boundary and the local structures should be bidirectional, especially in small particle systems.

### Effect of small rounded corners

The roundness ratios of real tetrahedral particles are often small. The effect of small rounded corners is especially concerned. Figure 4(b) gives the packing density of the def DC and the def QA (deformed quasicrystal approximant) families as a function of the roundness ratio *s*. Interestingly, an unusual increase of the packing density is first observed when the packed tetrahedra begin to round for both the def DC and def QA evolution families, as shown in Fig. 4(b). This unexpected phenomenon is unlike the dense packing of spherocubes (parallel cubes) in Ref. 37in which the packing density shows a continuous decrease trend with the increase of the roundness parameter, and differs from the dense packing of physical tetrahedra in Ref. 32in which the density decreases first when the tetrahedra begin to deform and the density does not exceed the densest ideal tetrahedron packing during the evolution from a tetrahedron to a sphere. Remarkably, the DC packing of ideal tetrahedra with a density of about 0.8563 is slightly denser than the QA packing with a density of about 0.8503, whereas the maximum density of the def QA family (Φ ≈ 0.8763) is larger than that of the def DC family (Φ ≈ 0.8704). The maximum value appears when the roundness ratio *s* is around 0.16 for both families, i.e., the spherotetrahedra with *s* ≈ 0.16 best fill space. This result is intriguing since the densest known packing of ideal tetrahedra is the DC structure. Despite the competition for the denser configuration from *s* = 0 to *s* = 0.32, the maximum densities of the two families are significantly larger than the densest known packing density of ideal tetrahedra.

**Figure 5:**
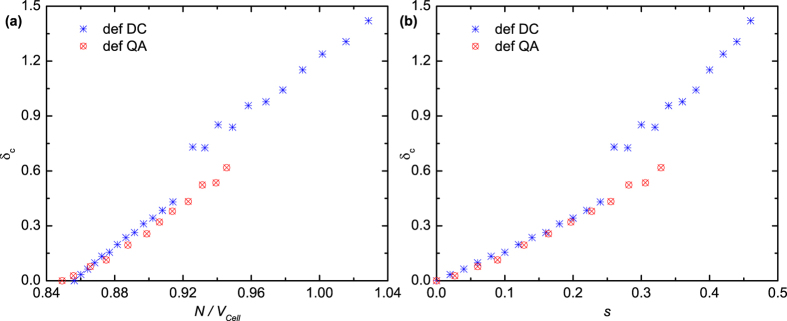
(**a**) The relationship between the local parameter δ_c_ and the global parameter *N/V*_*Cell*_ for the def DC and def QA families at small roundness ratios. (**b**) The relationship between δ_c_ and the roundness ratio *s* for the def DC and def QA families.

The local parameter δ_c_ still shows a linear relationship with the number density *N/V*_*Cell*_ for the def QA family, as plotted in Fig. 5(a). When *s* < 0.25, i.e., *N/V*_*Cell*_ < 0.92, the local parameter δ_c_ in Fig. 5(b) is nearly the same at a certain *s* for both the def DC and the def QA families, while the slope between the δ_c_ and the *N/V*_*Cell*_ of the def QA family is slightly smaller than that of the def DC family, as shown in Fig. 5(a). This is consistent with the appearance of the first peaks of Φ-*s* curves in Fig. 4(b) and the denser structure of the def QA.

## Discussion

In this work, the one-parameter shape of spherotetrahedra is studied to explore packing properties as a function of the roundness ratio *s*. The results present several structures during the evolution from ideal tetrahedra to spheres (Figs 2(a) and 4(b)). These structures can be further classified by studying the geometric variation of the packing unit cell (Fig. 2(b,c)). The observation that the packing density is discontinuous with the increase of roundness demonstrates that small deformation in the particle shape may lead to completely different dense packing structures, especially in the evolution from the def DC to the def FCC at *s* = 0.75, and in the evolution from the def FCC to the def HCP at *s* = 0.81 in Fig. 2. It can be seen that the face to face contact coming from the four plane surfaces of a particle, plays a dominative role when *s* is smaller than 0.75 according the illustration in Fig. 3, while the rounded corner has a more important impact in determining packing structures when *s* is larger than 0.75 and leads packing configurations to analogous structures of the FCC and the HCP arrangements.

The cylindrical and triangular surfaces account for a small percentage of the total surface of a spherotetrahedron when the roundness ratio is close to 1 (e.g., the spherotetrahedron model in Fig. 1(b) with *s* = 0.98). The particles are extremely similar to spheres and pack analogously to the HCP arrangement for the dominative effect of rounded vertexes. Yet, the existence of the cylindrical and triangular surfaces is not negligible, which makes highly rounded spherotetrahedra pack denser than the densest sphere packings (the right side of Fig. 2). The result is consistent with the Ulam’s packing conjecture^38^ that the optimal packing density for congruent sphere packings is smaller than that for any other convex body.

The results of this work demonstrate that spherotetrahedra with small rounded corners can pack more effectively than ideal tetrahedra. It is natural to raise the question which regular spherotetrahedra best fill space. The results in our simulation suggest that the spherotetrahedra with *s* ≈ 0.16 pack densest in space and the densest configuration is the def QA containing 82 particles. It is not known whether the def QA or the def DC can be densified by a compression process, or there are other structures of spherotetrahedra denser than the densest def QA. Additionally, it is worthwhile to explore the mechanism why the maximum densities of the def DC and def QA families, which are obtained from different packing protocols, both appear at *s* ≈ 0.16 (Fig. 4(b)). For real particle packings, sharp vertexes and edges are the most fragile parts of packed tetrahedral particles. According to the results of this work, spherotetrahedral particles with small rounded corners can pack even denser than ideal tetrahedra, but are not relatively easy to damage in the packing process. It provides additional substitutes for building block design. More generally, the rounded corner effect on other polyhedra deserves to be studied systemically, especially for the small rounded corner case, to find out whether the increase of packing density is a particular characteristic of tetrahedra or a more common phenomenon occurring at some other polyhedra.

In summary, we use the ASC^14^,^15^,^24^,^29^ and relaxation^25^,^26^,^27^ algorithms to simulate the evolution of the dense packings of spherotetrahedral particles. The results reveal diverse packing behaviors from the ideal tetrahedron packing to the sphere packing. The packing evolution with particle amount no larger than four can be classified into three families, i.e., def DC, def FCC, and def HCP, or further partitioned into seven regions according to the discontinuities of the packing unit cell. A good linear correlation between the local parameter δ_c_ and the global parameter (number density) *N/V*_*Cell*_ is observed in all the seven partitioned regions, which shows an interaction between the local packing structures of particles and the packing boundary. Furthermore, particles with small rounded corners can pack better than ideal tetrahedra, which have a best known density of about 0.8563. The maximum density of the deformed quasicrystal approximant family (Φ ≈ 0.8763) at *s* ≈ 0.16 is the densest configuration we obtained, which may suggest that the maximum density of the def QA for spherotetrahedra could exceed that of the def DC.

## Additional Information

**How to cite this article**: Jin, W. *et al.* Evolution of the dense packings of spherotetrahedral particles: from ideal tetrahedra to spheres. *Sci. Rep.* **5**, 15640; doi: 10.1038/srep15640 (2015).

## Supplementary Material

Supplementary Information

## References

[b1] Zallen R. The Physics of Amorphous Solids (Wiley, New York, 1983).

[b2] Torquato S. Random Heterogeneous Materials: Microstructure and Macroscopic Properties (Springer, New York, 2002).

[b3] Chaikin P. M. & Lubensky T. C. Principles of Condensed Matter Physics (Cambridge University Press, New York, 2000).

[b4] Edwards S. F. in Granular Matter (eds. Mehta A.) 121–140 (Springer, New York, 1994).

[b5] Aste T. & Weaire D. The Pursuit of Perfect Packing 2nd edition (Taylor & Francis, Boca Raton, Fla., 2008).

[b6] Chen E. R., Engel M. & Glotzer S. C. Dense crystalline dimer packings of regular tetrahedra. Discrete Comput. Geom. 44, 253–280 (2010).

[b7] Haji-Akbari A., Engel M., Keys A. S., Zheng X., Petschek R. G., Palffy-Muhoray P. & Glotzer S. C. Disordered, quasicrystalline and crystalline phases of densely packed tetrahedra. Nature (London) 462, 773–777 (2009). 20010683 10.1038/nature08641

[b8] Baule A. & Makse H. A. Fundamental challenges in packing problems: from spherical to non-spherical particles. Soft Matter 10, 4423–4429 (2014). 24898797 10.1039/c3sm52783b

[b9] Baule A., Mari R., Bo L., Portal L. & Makse H. A. Mean-field theory of random close packings of axisymmetric particles. Nature Commun. 4, 2194 (2013). 23877324 10.1038/ncomms3194

[b10] Van Anders G., Klotsa D., Ahmed N. K. & Engel Glotzer S. C. Understanding shape entropy through local dense packing. Proc. Natl. Acad. Sci. USA. 111, E4812–E4821 (2014). 25344532 10.1073/pnas.1418159111 PMC4234574

[b11] Haji-Akbari A., Engel M. & Glotzer S. C. Phase diagram of hard tetrahedra. J. Chem. Phys. 135, 194101 (2011). 22112060 10.1063/1.3651370

[b12] Chen E. R. A dense packing of regular tetrahedra. Discrete Comput. Geom. 40, 214–240 (2008).

[b13] Conway J. H. & Torquato S. Packing, tiling, and covering with tetrahedra. Proc. Natl. Acad. Sci. USA. 103, 10612–10617 (2006). 16818891 10.1073/pnas.0601389103 PMC1502280

[b14] Torquato S. & Jiao Y. Dense packings of the Platonic and Archimedean solids. Nature (London) 460, 876–879 (2009). 19675649 10.1038/nature08239

[b15] Torquato S. & Jiao Y. Dense packings of polyhedral: Platonic and Archimedean solids. Phys. Rev. E 80, 041104 (2009). 10.1103/PhysRevE.80.041104 19905270

[b16] Kallus Y., Elser V. & Gravel S. Dense periodic packings of tetrahedra with small repeating units. Discrete Comput. Geom. 44, 245–252 (2010).

[b17] Betke U. & Henk M. Densest lattice packings of 3-polytopes. Comput. Geom. 16, 157–186 (2000).

[b18] Baker J. & Kudrolli A. Maximum and minimum stable random packings of Platonic solids. Phys. Rev. E 82, 061304 (2010). 10.1103/PhysRevE.82.061304 21230669

[b19] Jaoshvili A., Esakia A., Porrati M. & Chaikin P. M. Experiments on the random packing of tetrahedral dice. Phys. Rev. Lett. 104, 185501 (2010). 20482187 10.1103/PhysRevLett.104.185501

[b20] Neudecker M., Ulrich S., Herminghaus S. & Schröter M. Jammed frictional tetrahedra are hyperstatic. Phys. Rev. Lett. 111, 028001 (2013). 23889445 10.1103/PhysRevLett.111.028001

[b21] Smith K. C., Alam M. & Fisher T. S. Athermal jamming of soft frictionless Platonic solids. Phys. Rev. E 82, 051304 (2010). 10.1103/PhysRevE.82.051304 21230470

[b22] Smith K. C., Fisher T. S. & Alam M. Isostaticity of constraints in amorphous jammed systems of soft frictionless Platonic solids. Phys. Rev. E 84, 030301 (2011). 10.1103/PhysRevE.84.030301 22060320

[b23] Smith K. C., Srivastava I., Fisher T. S. & Alam M. Variable-cell method for stress-controlled jamming of athermal, frictionless grains. Phys. Rev. E 89, 042203 (2014). 10.1103/PhysRevE.89.042203 24827237

[b24] Jiao Y. & Torquato S. Maximally random jammed packings of Platonic solids: hyperuniform long-range correlations and isostaticity. Phys. Rev. E. 84, 041309 (2011). 10.1103/PhysRevE.84.041309 22181137

[b25] Zhao J., Li S., Jin W. & Zhou X. Shape effects on the random-packing density of tetrahedral particles. Phys. Rev. E 86, 031307 (2012). 10.1103/PhysRevE.86.031307 23030912

[b26] Li S., Lu P., Jin W. & Meng L. Quasi-random packing of tetrahedra. Soft Matter 9, 9298–9302 (2013).

[b27] Jin W., Lu P., Liu L. & Li S. Cluster and constraint analysis in tetrahedron packings. Phys. Rev. E 91, 042203 (2015). 10.1103/PhysRevE.91.042203 25974480

[b28] Jiao Y. & Torquato S. Communication: a packing of truncated tetrahedra that nearly fills all of space and its melting properties. J. Chem. Phys. 135, 151101 (2011) 22029288 10.1063/1.3653938

[b29] Chen D., Jiao Y. & Torquato S. Equilibrium phase behavior and maximally random jammed state of truncated tetrahedra. J. Phys. Chem. B 118, 7981–7992 (2014). 24716833 10.1021/jp5010133

[b30] Damasceno P. F., Engel M. & Glotzer S. C. Crystalline assemblies and densest packings of a family of truncated tetrahedra and the role of directional entropic forces. ACS Nano 6, 609–614 (2012). 22098586 10.1021/nn204012y

[b31] Chen E. R., Klotsa D., Engel M., Damasceno P. F. & Glotzer S. C. Complexity in surfaces of densest packings for families of polyhedral. Phys. Rev. X 4, 011024 (2014).

[b32] Kallus Y. & Elser V. Dense-packing crystal structures of physical tetrahedra. Phys. Rev. E 83, 036703 (2011). 10.1103/PhysRevE.83.036703 21517621

[b33] Kim F., Connor S., Song H., Kuykendall T. & Yang P. Platonic gold nanocrystals. Angew. Chem. Int. Ed 43, 3673–3677 (2004). 10.1002/anie.200454216 15248270

[b34] Greyson E. C., Barton J. E. & Odom T. W. Tetrahedral zinc blende tin sulfide nano- and microcrystals. Small 2, 368–371 (2006). 17193052 10.1002/smll.200500460

[b35] Tsuji M., Tang X., Matsunaga M., Maeda Y. & Watanabe M. Shape evolution of flag types of silver nanostructures from nanorod seeds in PVP-assisted DMF solution. Crystal Growth & Design 10, 5238–5243 (2010).

[b36] Gantapara A. P., de Graaf J., van Roij R. & Dijkstra M. Phase diagram and structural diversity of a family of truncated cubes: degenerate close-packed structures and vacancy-rich states. Phys. Rev. Lett. 111, 015501 (2013). 23863011 10.1103/PhysRevLett.111.015501

[b37] Marechal M., Zimmermann U. & Löwen H. Freezing of parallel hard cubes with rounded edges. J. Chem. Phys. 136, 144506 (2012). 22502532 10.1063/1.3699086

[b38] Gardner M. in The Colossal Book of Mathematics: Classic Puzzles. Paradoxes, and Problems 135 (Norton, New York, 2001).

