# Supplementary material for: Evolution of the dense packings of spherotetrahedral particles: from ideal tetrahedra to spheres
Source: Sci Rep. 2015 Oct 22;5:15640. doi: 10.1038/srep15640 (PMC4614866; doi:10.1038/srep15640)
Supplement: Supplementary Information [file srep15640-s1.pdf]

Evolution of the dense packings of spherotetrahedral particles: from ideal tetrahedra to spheres

Weiwei Jin, Peng Lu & Shuixiang Li*

Department of Mechanics and Engineering Science, College of Engineering, Peking University, Beijing 100871, China

Configuration data of the densest spherotetrahedron packing we obtained.

In the following, we list the coordinates of the four vertexes of the inner ideal tetrahedron belonging to each spherotetrahedron in the densest packing we obtained. The packing density of the structure is 0.8763. A spherotetrahedron can be mathematically treated as a set of points with the distance to an inner tetrahedron smaller than a given value *R*. In the first line, the edge length *L* of the inner tetrahedron and the value *R* are given. The dimensions of the cuboid cell and the number of particles are listed in the next two lines, and following are the coordinates of the four vertexes of the inner ideal tetrahedron belonging to each spherotetrahedron in the 82-particle system.

10.40962765	0.416385106	
43.15659709	43.15659709	11.14000000
82		
13.6273369300391210	36.3695420191048560	3.3753864526872603
9.9838610558645744	37.6355345865535540	13.0440313517773120
5.9767117288871905	43.1876764250638490	5.2033156263617153
4.1016068190476709	32.9678204056922300	5.8348790278701497
11.2987222440364410	25.4464497416251340	0.1020418310374509
5.0406753307471348	20.4409172696826540	6.7459745003875131
14.9531119682490840	17.4486443377565090	5.6734772185111435
12.7176495513551120	26.4688925073945140	10.3636988073002850
28.4021010387477130	14.6237737286336780	3.6679729073414471
32.1702520232178490	20.3439633504820670	-4.1704463631592477
35.8463433139832900	21.6713892169542500	5.4775918416826661
38.0804284578540830	12.9772042534354990	0.2068841851079015
41.3586403227002460	24.8915633230637110	6.9838742254738175
44.7320329837675670	34.6821567427847200	5.9232846463483781
35.1732321196066380	32.8412879210951730	9.6112211739750162
43.5147209936586420	30.5964275987441990	15.4198796514689410
4.6684565968670295	46.9539780082712430	5.7563084499626935
-1.1601421025220331	42.4249671101971370	13.0963251596714990
-4.9794757683780260	43.7587856783605030	3.5049766523919050
2.5572386034062040	36.7768972942794220	5.1817350973775849
34.6688879729831360	32.3613925723331520	-0.7684589792424941
33.5197356280111900	33.4823471911464590	9.5166398264101062

43.0536220988556270	32.9266775854665500	5.3745886604445596
37.1778464645875250	41.3734735437182910	3.7974879831704555
15.7317984395760230	36.2047827737280630	3.6894092332939108
21.5461579966272970	35.2205054029831710	12.2675657258475820
14.3598062027488390	28.0394138598222010	9.9985086628409441
22.9940802353724470	28.7665122365149520	4.2295410176768771
37.9057512724952730	19.5619692346992370	5.8160974873674895
47.6214794731161390	15.9334976264688940	6.7100589691940522
39.6260144848541240	10.3557321610672640	10.3599109997713120
40.9698977567388030	11.4246342719963430	0.0928873882611425
21.5454514906544820	27.5274649257979900	3.6034539523125657
14.7867114093397680	35.4435448567857920	3.7277875773811120
11.9432165204276850	26.2331781031336750	-0.2021015928727266
13.3779132097788050	27.2970467777528540	10.0531495821327330
29.6518611370541550	43.2174939865487320	12.0747563962214600
32.5655134334480460	33.5188241031333760	9.6650301464836730
26.4650768825847710	38.8886321318718520	3.1604022990598466
36.4465586834198090	41.5895959020007810	4.3583854299175684
4.4551063574508296	16.7999812433709260	5.4343223709522288
6.8954500477874214	6.6992054240665970	4.8183691800497614
-1.2622391969959164	10.4149836939857200	-0.4736480011285649
8.4113942339346046	12.9294829518638590	-3.3819907488294909
27.5140495036309470	13.1988470591778690	14.4166030336503570
24.4678084071016890	23.1520419351288990	14.2954636314264220
31.0009075070428220	19.6192730545135770	7.0017328990038825
21.0470853226422250	16.5726959919607140	6.9899035330949797
17.7783475309477660	5.7562849996475833	-0.9351612487248393
25.7207822350946240	10.6956316390039770	3.6344047654818232
15.9523433271255200	13.8710793732103280	5.3239256442219620
19.2587621245802400	4.8494433963691765	9.3286761792803823
33.6157005395439440	32.6224132019208070	9.3198790201545521
35.0948293525088740	31.5022083391016920	-0.9230535106836264
43.2652423720284740	31.7076376204804550	5.5238447909437367
36.8617475346593790	23.5006366649412750	5.4966971613693927
23.0061985405058740	30.1622408065543870	2.5875546329478256
21.9916078215285000	35.5557670325152910	11.4329392110568030
25.6557378871200510	40.2247533325678290	2.8810350017813744
15.6331227456405650	37.4638588723347820	3.4148845290952217
33.0289766745539310	31.6857087364867490	9.3204039529710379
26.5171574087619350	25.8283569308550580	3.6947495478930947
34.4731570034802670	30.6845963852957620	-0.9398331324597682
36.2727882441342050	22.6124203085921920	5.3816895160103977
-1.3127640001934529	11.3096512770860510	10.4457912686829760
4.6093898040044037	17.6718718745940540	16.1738442712114970

8.3308116531176495	13.6676939090911700	7.3150674903389739
0.8484495644853953	20.8986828953342790	7.0194263780283794
8.1846857935763122	15.0269043858904330	-2.5443348373962356
5.1085920244480789	19.6540663313869180	6.2583560731006349
15.0393356104329480	16.7574643436270880	5.0962990324665016
11.2665688206292780	24.7398486462123830	-0.4180567239138879
47.2918119982125450	18.2758667609044250	6.3297924290605039
44.4265893075921670	28.2833375926909270	6.2927209353455984
43.5113199029412100	22.5752474706721670	-2.3640877657567581
37.2110036972841840	20.8013990554944110	5.7303439820815143
17.0874278656629240	5.1751849603059554	-0.8959273254728193
18.6170802649148190	4.1033834389820409	9.3447637082570232
15.3077924902182690	13.1932022273218180	5.4997465911101822
8.9464689694189943	4.9538944826413172	5.5876030851600831
36.5120331691166840	0.2454018885168791	4.8200965137960914
40.5389660993104780	-1.3407016360895314	14.2873260237879370
37.1774611856937940	8.1323199290865507	11.5813151308815420
30.3421930744542580	0.4502773843309009	13.2017101441183800
16.0913399385332310	6.2477015820307038	-0.6140598602285072
14.9624746868766410	14.6184258558543940	5.4700987657964841
9.0581951893107142	13.5305619398711910	-3.0338052509526712
7.5229526813853678	7.3425656210254733	5.1949297557656804
2.0752434724873519	29.9532791241483520	4.7927867365288606
0.5158741072443831	23.7517964401328140	-3.4212456174959307
10.1927521377013510	26.4723449474304790	-0.7162511230950923
4.7659233500352309	19.9087091389710660	5.2694453354811071
-1.0987436907036456	41.1078863412025510	3.2929160837660341
2.7071764673447509	34.7906830479840680	-4.0533933375817872
8.8426714433919287	38.0479268765630890	3.6994391675049023
1.2151276993007405	31.2273983814312610	5.6128955402879726
19.9145633865589140	16.5005709950747810	6.3993144825083625
13.7665175519530100	23.9205622744889440	10.3370307799741710
23.3987087920861860	22.6215544308886790	14.0644720400655810
21.9948490997657300	26.5250425039441070	4.5171003264328204
5.7510285456056067	44.9514934678874170	6.0197601654667059
2.9573726579138331	34.9244040898861170	5.9043773297641398
9.3639830641362973	38.4576242705697600	13.3092437073230200
-0.6637684825857368	41.2511638968207710	13.3353796173440390
-3.0861731701694781	9.6486447423339960	10.4962195148642420
-1.7691273773765555	10.6990646050151100	0.2238119926152866
4.8995288460197317	15.2200807409801090	6.8154570894953173
4.8481220568695749	4.8652488088289916	5.7500178331151544
31.4936192628495350	20.7968443139768060	-3.9051058578248043
35.1886254321021990	22.1458212045953290	5.7327122857953547

25.5346384174158380	25.2469994392249010	3.3782322818044790
27.7442595586714230	15.0908192664989540	3.9526245891756946
40.2809162520611470	40.9772038238709650	14.6308682545493460
30.1940989704490900	42.8190376578621180	12.8352867142750600
36.9170122230418160	42.4798321652072350	4.8950269677168370
34.4176120850758590	33.7446971644141700	9.9754564061886271
20.2330971080232840	33.3784564819250060	12.6370682805089360
22.7786498417098140	27.2444571030568600	4.6211740252874591
30.2353487291243610	31.2770423247396890	10.6623863161781960
23.9871157742361550	23.8268874194118960	14.3792565909548260
29.2098443259912490	9.1144867578560227	4.6129208479424824
21.4456861036930490	5.5695804259649284	10.5721014970718660
31.2694672395255040	2.8196253405978924	12.6436755449051790
28.1274503654528940	12.5934801819546110	14.3640938994672100
10.5543131607785340	-4.5453559920444508	3.0354547012452651
16.4972730261050200	2.9412594868098747	-1.0867006236694503
6.8627545997709545	1.3634926306923456	-4.6987712558801062
8.2664912817392562	5.4610488719130998	4.7669513255964695
10.6211120041456300	25.9725538664681220	0.0960364000867928
3.8301842718666830	31.4563514371616860	5.7680389168505561
4.2578611987376078	21.1009924509278480	6.7396255184879239
12.0386824539915020	26.9939222961740450	10.3579878890826520
30.1141334036982540	9.0247570405985247	4.9359646842165699
28.9067354759354310	13.2401146288310230	14.3770079771392680
32.4254505512781110	3.4878989296945382	13.4425129333338480
38.4642857734412050	11.4908603318062090	10.6415442139716450
30.1285558599970860	0.8742311185647826	11.5577023969558250
26.4537742721452030	-3.4805170770857439	2.8460724549830370
29.3258285273876920	6.5250620378060935	2.8522968025224436
36.5281990580506670	-0.9577776599881972	3.5546618132168684
41.2597308805084500	12.0059480904558420	-0.4453773452476768
47.2523694651086570	17.4704020971285590	6.0805994410352655
44.0274746791098720	21.7500476317220870	-2.8438072915961703
37.2334459214937250	20.0131462306401810	4.8493516548948978
4.9522494766750267	18.4261195712174200	6.2716415077526326
15.0099874329233640	15.8159279188024920	5.6478189743051086
7.7356142640088779	8.3955070687862250	6.2652306843182357
8.6674015113841012	14.0610975211829570	-2.4176953871942914
21.8605606556370800	27.2932493255982610	4.8311372621332049
13.6322291018389410	24.6887691120165230	10.6510677143496670
23.2644203385226400	23.3897612624952770	14.3785089812874620
19.3911984667425090	32.9790753122202530	13.1937912093587960
15.3643689572014000	15.8840995084961440	15.4625903509784100
8.5073639751993468	15.2852721089390290	7.6534139389968177

12.2441449376025770	24.5433744911346810	10.6003371023564480
18.4269025282733030	17.7581219646134690	5.6917523229093243
1.9852397297541424	30.7757599693764590	5.5765878119112928
9.8499269884640235	37.2217530984132950	3.3503320507299699
10.0385438171902040	27.3985293665074360	-0.0890061147023826
3.3025900121493872	34.2105296426400050	-4.1613428376581219
10.6166841959595550	38.1057090617318580	13.4563235707091110
6.4834233122154021	43.7476113659492540	5.7462366277662413
16.1705705893358310	45.7071758336082180	9.0142073343939657
14.1285150059676030	36.9789152558514050	3.7219654566974016
44.9823787530395830	35.4218169880354380	-4.8171521740615608
41.2091510743690890	41.2926881466130170	2.9065757170723261
35.3927981471151720	33.6783780657255960	-1.1619213554061611
43.6913184568858380	31.3384419466885120	4.6707116210525577
4.4773153944138500	18.9212437255611730	16.1509174726440290
0.8088182796871876	23.0553026198202180	7.3298045162136436
10.5869432011759430	25.0066620519018910	10.3199244467073240
7.9696132894476879	15.5091354634140650	6.9573479573674124
19.7159039346864410	16.1020792200913160	-3.9942183712882757
23.6640429496449340	21.9651892703458030	3.6475410248991738
13.9025308840302060	23.5398043947474280	0.3927265579319572
16.2759410639021450	14.9434227400228680	5.7620369769901449
11.1905165830881240	26.5859316017559980	-0.0153670717308960
14.0340502143038910	35.7772819903601090	3.9587670897256446
4.4366311475559339	32.1193390056291650	5.6526821374005474
12.6138399799727980	27.6034284672554440	10.2461726980640790
18.2171594686559640	4.3920104448368278	-1.0926791129880780
16.1211417693647650	-4.7057666523027830	3.5113962020616771
25.9735777193356690	-1.4599886615627802	2.6426160815776676
19.4663553539869230	3.3522417213888378	9.1892826868381281
28.8382055465273840	7.6381473058466902	4.0834396948057723
30.5266541915331510	1.3690322041188168	12.2202501771994230
36.3451229628541130	0.4403190235435215	3.6386850054791346
37.4603561475005120	8.7494628923812794	9.8091575093960657
22.4057465497516370	37.0165129012646330	13.2509468050434760
19.9837400353824960	46.5711269415340890	9.9037714589296879
29.6668478494445370	44.4739383208077330	13.0974481429971790
26.1467126821922380	40.4522700767430270	4.1646333150199872
20.4410781804407690	15.4032382942288990	5.9829477507599327
20.5612462535265690	5.5526908983810994	9.3463721140684477
28.4509277006907130	9.1915007658810115	3.6129318535378419
27.1578183025250370	12.5149645571617610	13.3926457717969430
-1.1975989402144349	9.4844339502054282	-0.0424974155616304
6.8493765280177810	5.8768402961851152	5.4884881795514247

-1.1925671078312563	-0.3670658950969125	3.3202801998589058
5.3227110218010125	2.5497252464403539	-4.2562512058589936
12.1546699416928910	25.4183030093440400	-0.1537259705469411
13.5601160014751440	26.4796580551895000	10.1058351629185700
16.6556727216510510	17.7796575068869740	5.3008516184307402
21.7457852523568920	26.7139562232925480	3.6793064658049683
21.6566940116574250	37.0037750982311950	12.7679057185573730
17.6162095992896890	46.1559199900231080	9.8916099405413860
14.8545919575936680	37.4482327379154610	4.9006182023105920
11.6369494328444320	39.0956134069936000	14.6624449434299110
39.2472395135628180	9.2621139362352416	10.1881221301922410
47.1815347429281500	4.4787179994022086	5.4419204555350191
37.4290981805504970	1.2020272172716997	3.8564784267662890
40.5642853094226870	10.3125337892342370	-0.0842853926513865
28.2084276241369130	8.2739397290776147	4.4995946584686211
26.0184576313321120	-1.8742345514910836	3.7387148960291428
29.8802053498615980	1.9661736438494326	12.6099333727983800
20.1465589929758760	4.2820834703244568	9.7371232354018673
26.5415368674558000	11.3167498489673370	3.3651210269273495
16.9086309120205560	14.3985138299848290	5.8288989046893827
24.3008415179507370	21.4684309500923230	3.8971012744752693
20.4668428658628020	15.6920267161719660	-3.8678170299346548
38.7443505269404510	10.6127033596710110	9.9937282658744575
30.4690609306310480	10.8036983452083390	3.6815613777802541
35.9043124949537430	19.5131982446741610	5.4028979935570813
40.0482256111767400	11.8260171898577650	-0.2623980556934793
32.3820505811621120	20.9552330744123370	-4.9316012499067527
27.0036084147430270	26.2894442850409500	2.2083496206778364
36.2550098847495830	22.1902857438148760	4.6514660538283827
35.7113608041304500	30.3177217725539630	-1.8300134907700674
30.2907591985891960	9.9468344236219028	3.7409147258020519
39.8935178984755860	10.9643490973042380	-0.1465056337621727
38.5288115136952670	9.7482229756019887	10.1013703193758780
36.7539294005888170	1.7874102192491090	3.6333256724821714
24.9080902256995850	23.9278883401791300	13.4072970635468100
31.5544377490500560	30.7210455801583570	9.1600123870555326
31.4278435477110170	20.8341732617294930	5.9051579389673403
23.4909169740670120	27.1690440498187830	3.6171552131148594
19.1896079366765340	45.9605869682040340	9.9615190479796389
25.4034189941008290	40.9045090577463740	3.3143409912627462
22.3305014669280380	36.2727293209120700	12.1157125975652490
15.4888810156683300	37.9426368374757740	4.4499454279568393
25.1020436685209210	10.5632486862153790	2.1071481809800363
19.4229106159039700	15.6024874958974780	-5.0142188086267998

16.4750383767338950	6.1762149358360565	-1.7254830794826255
15.7545004472123970	14.3736011520350680	4.6497841403856208
31.8050417924298400	31.7378468316479500	9.7706641789686426
23.6787475183091840	28.0640495633357540	4.4015950964199462
22.1162921433408750	34.2769398782621830	12.6064153227890370
26.1578421396153860	38.1581980807790660	3.8336132502291904
17.3582246489442650	4.3261478193136584	-0.8186084409782417
18.5973021366468280	3.2545023441875802	9.4613045336952002
9.0277822889015926	3.8081952865452067	5.4019866327592005
14.8729114791480780	-4.6579675288658970	3.8148824320390453
28.7020633259914920	13.9949944663042060	14.0208715823300910
29.9281655896386560	9.7803926034597133	4.5819017133469249
32.3686970610778670	19.7733815846964020	6.1769958073396003
38.2670592716799970	12.2527224631759180	10.3012360307864640
32.5589813029332400	32.8870465692059640	9.0670395554463870
25.7819515734170360	37.4065517438651800	2.5858136812742374
35.4956747287112010	41.0711773983429400	3.3437378767359083
33.7402873969618260	31.8847516318550710	-1.2266608764244866
41.1816256353095510	-0.5364856335178008	14.0910213898994600
46.4408418253275030	4.8837469315703235	6.9270789125470875
37.2116065341249040	0.6980497323413895	4.5476876653394518
37.7274120631945780	8.8034886768293283	11.0589204199591680
14.0436053815728600	28.1165559225696950	11.0317305246211190
10.7424062177385840	37.4518826429395940	14.2432888560638130
14.6132389699256180	36.3183181501403570	4.6468294486962654
5.3489179093809227	32.2189062036028560	7.0400542170349425
2.5358291801232697	34.2063136384312400	5.9166547521332387
-1.0179560415170201	24.5629893827004080	7.5711201368007544
8.9080373252599845	26.8850769713872570	9.6789419331101776
1.2863665207879253	30.7172774631094790	15.6442349981260130
31.4661065030543450	19.4934588867405520	5.9073472929873851
29.2586206622808370	9.6042969265157101	3.5215916897604962
27.7552355707370530	13.0793254348078700	13.2182074882969010
21.5592369652114830	16.3225608360401540	5.5077358280627839
37.7105212864252760	43.0756049117328250	3.6942942077393384
45.2472163324289840	36.1728218111538240	5.6717069267085831
41.3356572464314880	41.9616840476322750	13.3885150320916470
35.5698900282849310	34.4352936450756180	9.0908733874649315
8.5800113435590291	6.1507312206127400	15.4026767150581580
16.8275806575303410	3.6395966685124659	9.5689685047099271
7.2034964305002864	2.0517541173062597	5.9335721527477450
10.8582226241839200	11.7479836558227400	6.9267775681513442
25.5518893896159620	36.5508079544454890	2.3812390939975101
25.6502068365081330	26.2016621032341880	3.4974208182916415

33.5608782672884520	30.9972361815624670	-1.2758479342495195
32.2436567199118610	32.0921244548673600	8.9918925608559785
39.3411024779913630	12.2159776868397980	10.3918640370627800
36.7420250552972120	20.9215502889421820	15.4730204046268070
43.4697110571013570	21.3365103927398430	7.5404043806286953
33.4053395783859200	19.3867824830326970	5.7328270995229618
37.2398033980914530	22.0103583177180230	5.5429941729822252
43.0893712050760200	23.0586033138102490	-3.0035891625315267
35.9874788525285110	30.2922933720528780	-0.6377487272640283
44.5962250847801440	29.3643674409881540	5.1405546210852098
18.4312856478006780	5.2567345648864610	-1.2794959701705899
26.3011617265064790	10.0994569429182200	3.5135655029490098
19.7082944057479850	4.1981903163332630	8.9971319065603712
26.4472012267858680	-0.2524987576883726	2.4291147451036874
18.8241975926964540	16.9506738972278300	6.0002325471616995
8.7721352244576156	14.5375835754907500	7.2222738950178531
16.0771057831174030	7.2462867196007048	8.5768007895970158
15.0687838768217300	14.9532325785575820	15.5011505982073580
22.6861625649275140	26.7380169564546150	3.5322339419517372
20.7729328833207880	16.8045960450566480	5.9872388286453457
30.5805066564713460	20.2698110575583500	5.5821099443835891
24.2738559007788870	23.3813390829843120	13.2570625438522110
-0.1868640095791272	-1.1891046927450475	3.6940954073706900
5.9588852324588224	2.0163792178041069	-4.0721769909442012
7.6109244519438803	5.4321235970673678	5.6213159071071397
9.6778583303041774	-4.4712239619868877	3.1694106727237297
0.0352562415266227	10.0538399291831570	9.8187717293401047
6.1066770096637626	2.5562378834169963	5.9092754407382406
10.0061094821251950	12.0586752874118460	7.5999728081285713
7.5992732528191080	5.9124772561245482	15.6493042671703360
36.3040636609876160	29.7184487691929360	8.8334351414427985
33.0736680776000470	20.2120325905507700	6.0851732134937162
43.2400206162714370	22.1179481830170060	7.2569708173636780
37.0047340709063590	21.8284554366519220	15.5875016202279540
15.4717323840400280	36.4581956900337830	4.6984818540354567
11.5109954843655160	38.2141191849968180	14.1636624493239990
14.6477785143383950	28.6448601800500630	11.5273350549887130
21.6610010743698180	36.1824043938952470	13.0637218416207880
25.8988503010598770	39.1290706750818630	4.1528751485252151
29.7096857849934080	42.2987332251161380	13.3066241097464530
22.2915493219167720	35.0022175886398510	13.0025537336847370
31.9286741397626590	32.7648546804919430	9.7652121893936226
